# Maintaining Low Prevalence of *Schistosoma mansoni*: Modeling the Effect of Less Frequent Treatment

**DOI:** 10.1093/cid/ciab246

**Published:** 2021-06-14

**Authors:** Diepreye Ayabina, Klodeta Kura, Jaspreet Toor, Matt Graham, Roy M Anderson, T Deirdre Hollingsworth

**Affiliations:** 1 Big Data Institute, Li Ka Shing Centre for Health Information and Discovery, University of Oxford, Oxford, United Kingdom; 2 London Centre for Neglected Tropical Disease Research, London, United Kingdom; 3 Department of Infectious Disease Epidemiology, School of Public Health, Faculty of Medicine, St Mary’s Campus, Imperial College London, London, United Kingdom; 4 MRC Centre for Global Infectious Disease Analysis, London,United Kingdom; 5 Abdul Latif Jameel Institute for Disease and Emergency Analytics (J-IDEA), School of Public Health, Imperial College London, London, United Kingdom; 6 Centre for Mathematical Modelling of Infectious Disease, London School of Hygiene and Tropical Medicine, London, United Kingdom; 7 The DeWorm3 Project, The Natural History Museum of London, London, United Kingdom

**Keywords:** schistosomiasis, modeling, morbidity control, EPHP, MDA

## Abstract

**Background:**

The World Health Organization previously set goals of controlling morbidity due to schistosomiasis by 2020 and attaining elimination as a public health problem (EPHP) by 2025 (now adjusted to 2030 in the new neglected tropical diseases roadmap). As these milestones are reached, it is important that programs reassess their treatment strategies to either maintain these goals or progress from morbidity control to EPHP and ultimately to interruption of transmission. In this study, we consider different mass drug administration (MDA) strategies to maintain the goals.

**Methods:**

We used 2 independently developed, individual-based stochastic models of schistosomiasis transmission to assess the optimal treatment strategy of a multiyear program to maintain the morbidity control and the EPHP goals.

**Results:**

We found that, in moderate-prevalence settings, once the morbidity control and EPHP goals are reached it may be possible to maintain the goals using less frequent MDAs than those that are required to achieve the goals. On the other hand, in some high-transmission settings, if control efforts are reduced after achieving the goals, particularly the morbidity control goal, there is a high chance of recrudescence.

**Conclusions:**

To reduce the risk of recrudescence after the goals are achieved, programs have to re-evaluate their strategies and decide to either maintain these goals with reduced efforts where feasible or continue with at least the same efforts required to reach the goals.

Schistosomiasis remains a public health problem in many countries, affecting over 230 million people around the world [1]. It manifests in 2 forms: intestinal or urogenital caused mainly by infection with *Schistosoma mansoni, Schistosoma haematobium*, and *Schistosoma japonicum*. In areas where schistosomiasis is endemic, significant morbidity could lead to complications in multiple human organ systems [2]. Schistosomiasis transmission requires contamination of water by feces or urine containing eggs, an intermediate snail host, and human contact with water inhabited by the intermediate host snail.

The World Health Organization (WHO) has set guidelines to control the morbidity induced by infection. The goals set are morbidity control and elimination as a public health problem (EPHP), achieved when the heavy-intensity prevalence in school-aged children (SAC; 5–14 years of age) is reduced to less than 5% and 1%, respectively [3]. For *S. mansoni*, a heavy-intensity infection is defined as 400 or more eggs per gram of stool and is usually assessed via the Kato-Katz method on 2 separate stool samples per individual [4]. The goals were set to be achieved in 2020 and 2025, respectively. However, moving towards post-2020 goals, WHO has proposed new timelines for the EPHP goal, now set to be achieved by 2030 [5]. The WHO end goal for schistosomiasis is interruption of transmission (IOT), defined as incidence of new infections reduced to zero [6]. The prevalence of schistosomiasis is usually highest in SAC, and the WHO treatment guidelines call for preventive chemotherapy that mainly targets SAC in endemic areas through periodic mass drug administration (MDA) [7]. In some high-transmission settings, WHO guidelines recommend treatment of adults at risk [8].

The success of schistosomiasis control programs in attaining morbidity control in countries like Japan and Brazil has led to greater impetus towards elimination of transmission [3, 9–11]. However, following achievement of either morbidity control or EPHP, infections may remain in the population and this could lead to resurgence if treatment programs are halted. Also, individuals not targeted for treatment, such as pre-SAC and adults, may remain a reservoir of infection. To prevent resurgence, after these goals are achieved programs will need to reassess their treatment strategies to either maintain these goals or progress from morbidity control to EPHP and ultimately IOT.

Previous modeling work suggests that in order to maintain the gains of achieving these goals, treatment will still be needed to curb resurgence [12]. Due to limited praziquantel (PZQ) supply, it is important to determine if we can maintain schistosomiasis at low levels with reduced efforts in terms of treatment coverage and frequency. In this study, we investigate whether with reduced MDA efforts (both frequency and coverage), the morbidity control and EPHP goals can be maintained without a high risk of resurgence above the WHO-defined threshold criteria.

## METHODS

We used 2 individual-based stochastic models of schistosomiasis transmission and control developed independently by Imperial College London and University of Oxford (ICL [13–15] and SCHISTOX [16], respectively). Both models are similar in terms of human host demography and age-dependent force of infection resulting in an age profile of infection but differ in the processes describing egg production. While the mean number of eggs produced is proportional to the number of worm pairs (male and female worm pairs) in SCHISTOX, it is a function of the number of female worms and monogamous sexual reproduction in the ICL model.

We focused on a single community with a population size of 500 without migration. We considered 2 age profiles of infection with low and high burdens of infection in adults (produced by varying the age-specific contact rates [17]). For each age profile of infection, we simulate moderate (10–50% baseline SAC prevalence) to high (≥50% baseline SAC prevalence) transmission settings and administer treatment annually for a variety of age-group coverage levels until both goals are achieved. Intervention coverage is assumed to be random at each round of treatment. The basic reproduction number *R_0_* was varied in the ICL model, and the overall contact rate *β* was varied in SCHISTOX to simulate these ranges of baseline prevalence settings. The parameters used for this study are outlined in Supplementary Table 1. For every scenario, we run 500 model iterations and define a goal as achieved if at least 90% of the simulations are below the required threshold. All analyses were performed in accordance with the PRIME-NTD criteria (Supplementary Table 2).

### Maintenance Strategies

The simulations are divided in 2 phases: first, achieving both goals and then maintaining each of them. Previous work has suggested what treatment frequencies and coverage are required to achieve both goals [18, 19] using deterministic models. Building on this, for the first part of the simulations, we considered meeting both goals within a 10-year program with treatment administered annually at 75% SAC coverage regardless of the transmission setting, without systematic noncompliance [20]. In the 10th year, the endpoint of SAC heavy-intensity infection prevalence was evaluated to determine whether the morbidity control and EPHP goals had been met. Where either goal was not achieved, we adjusted the treatment strategies to a wider coverage (including increasing SAC coverage and/or including adults) such that both goals are met within 10 years.

**Figure 1.**
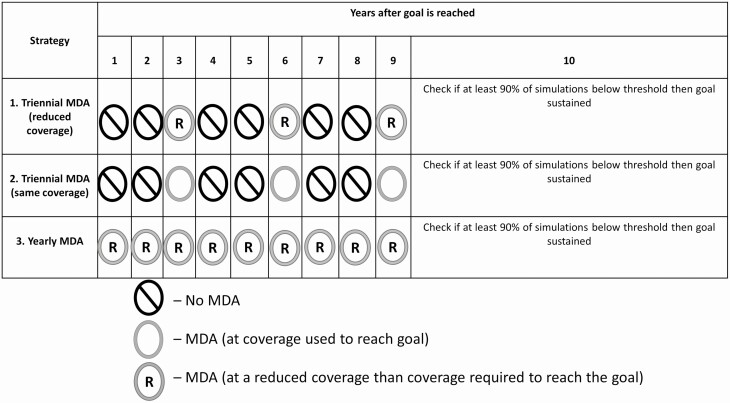
Maintenance strategies investigated in this study. For strategies 1 and 3, we initially applied a reduced coverage of 50% of SAC only in all settings. If, in the 10th year, the prevalence of heavy intensity was above the threshold of either goal, we increased this coverage and/or included adults while ensuring that we stayed below the coverage required to reach the goals. Abbreviations: MDA, mass drug administration; SAC, school-aged children.

Once the goals are reached, we consider another 10-year maintenance program with different treatment schemes for maintaining each goal (Figure 1). Each scheme involves reduced effort (either in terms of coverage and/or frequency of treatment) compared with the MDA strategies that were required to reach the goals. The maintenance strategies considered are as follows:

1. Triennial MDA (reduced coverage): This strategy involves stopping MDA for 2 years as soon as goals are met, followed by 1 round of treatment at a reduced coverage than what is required to reach the goal in the first part of the simulations. Altogether there are 3 MDA rounds over 9 years.
2. Triennial MDA (same coverage): This also involves stopping MDA for 2 years as soon as goals are met, followed by 1 round of treatment at the same coverage required to reach the goal in the first part of the simulations. Altogether there are 3 MDA rounds over 9 years.
3. Yearly MDA (reduced coverage): This strategy involves continuing annual MDA for 9 years but at a reduced coverage than what is required to reach the goals in the first part of the simulations.

In the 10th year, we calculate the probability (proportion of simulations) of remaining below the WHO-defined criteria for each of the goals. For the reduced coverage maintenance strategies, we initially applied a reduced coverage of 50% of SAC in all transmission settings. We increased this reduced coverage if, in the 10th year, the probability of being below the thresholds is less than 0.9 while ensuring we stayed below the coverage required to reach the goals.

## RESULTS

### Reaching the Goals

In moderate-prevalence settings (baseline prevalence in SAC <50%), results from both models suggest that with annual treatment of 75% of SAC only (with no systematic noncompliance), morbidity control can be achieved within 3 years, regardless of the burden of infection in adults. Results from both models suggest that EPHP can be achieved within 5 years in a low-adult-burden setting and within 8 years in a high-adult-burden setting without systematic noncompliance (Supplementary Figure 1).

In high-prevalence settings, once the baseline SAC prevalence is above a cutoff point, treatment of SAC and adults is required for the EPHP goal to be met within 10 years [18, 19, 21]. The cutoff baseline SAC prevalence varies with the age profile of infection and across the 2 models used in this analysis. For simplicity, we will refer to the cutoff baseline SAC prevalence as *C_l_* and *C_h_* for low- and high-adult-burden infection settings, respectively. Results suggest that *C_l_* is 76% and 67% for SCHISTOX and ICL models, respectively, while *C_h_* is 58% and 53% for SCHISTOX and ICL models, respectively. For settings with baseline SAC prevalence above this, intensified treatment is needed, such as higher coverage of SAC and/or an expansion in treatment coverage to include adults (Supplementary Figure 1).

### Maintaining the Goals

Even though the goals are reached, there is likely to be residual infection (often lower intensity) in SAC and/or non-SAC, which could lead to resurgence if treatment is halted. There is a higher risk of resurgence in high-transmission settings, and this is escalated if control efforts are reduced before the prevalence falls to low levels.

**Figure 2.**
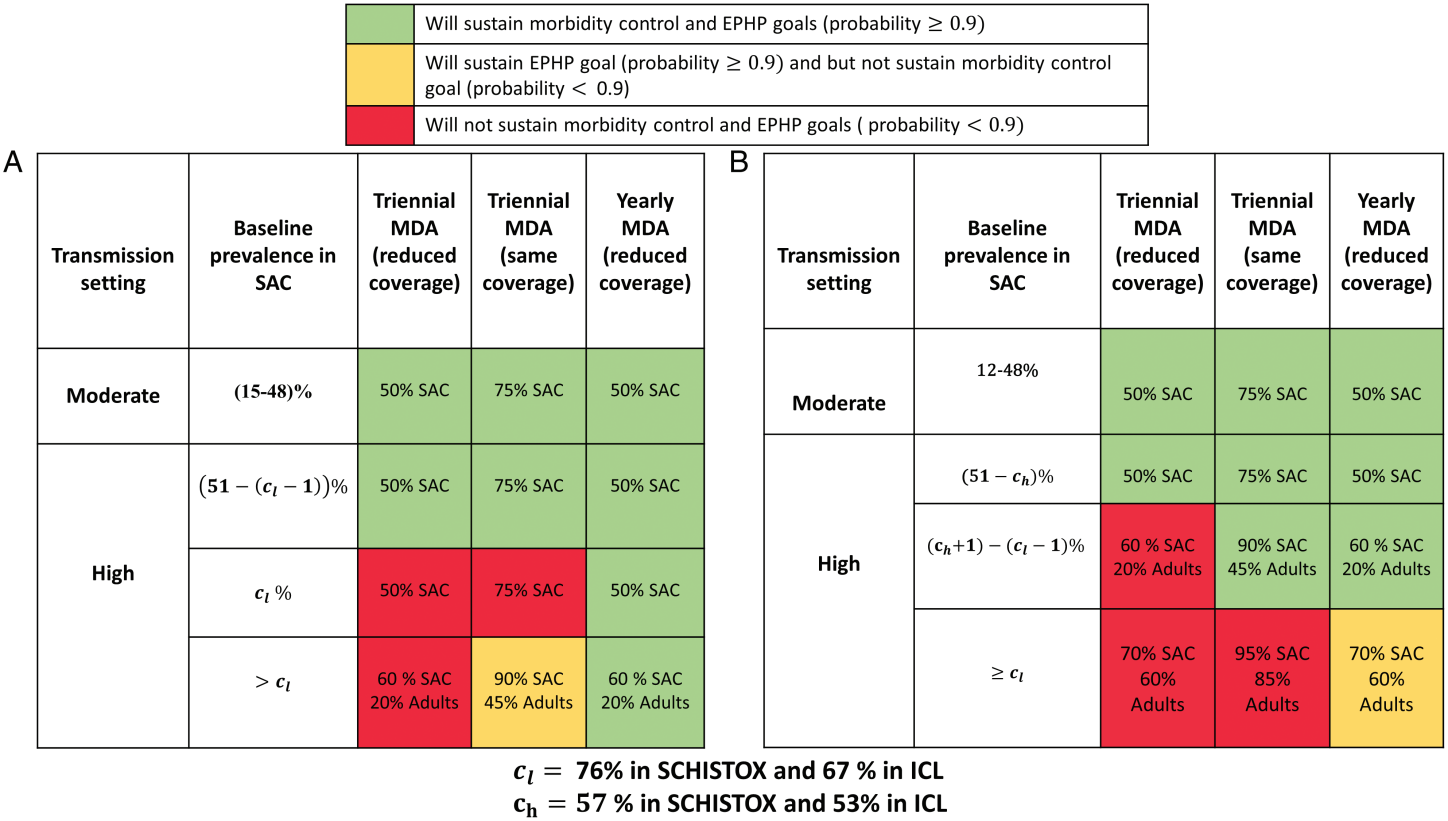
Projected outcomes for *Schistosoma mansoni* employing different maintenance strategies for moderate- to high-transmission settings with (*A*) low and (*B*) high adult burden of infection with coverage levels used in each scenario. Abbreviations: EPHP, elimination as a public health problem; MDA, mass drug administration; SAC, school-aged children.

For baseline SAC prevalence less than the cutoff prevalence, regardless of the burden of infection in adults, both goals can be maintained using any of the 3 strategies (Figure 2). Above the cutoff baseline SAC prevalence, maintaining the goals depends on the strategy employed and the transmission setting, with more efforts required in settings with a high burden of infection in adults (Figure 2).

Regardless of the burden of infection in adults, for transmission settings with high baseline SAC prevalence (≥ *C_l_*), there is a higher likelihood of maintaining the EPHP goal than the morbidity control goal (Figure 2). In these settings, adopting a maintenance strategy with a reduced coverage of 50% of SAC is not sufficient to maintain the goals even if MDA is not stopped after the goals are reached (Supplementary Figure 3). Adopting the yearly MDA strategy and increasing the coverage close enough to what is required to meet the goal increases the likelihood of maintaining the goals. Specifically, for settings with baseline SAC prevalence greater than 50%, continuing MDA with a reduced coverage of 60% of SAC and 20% of adults is sufficient to maintain both goals in low-adult-burden settings, while 70% of SAC and 60% of adults is sufficient to maintain the EPHP goal alone in high-adult-burden settings (Figure 2).

## Discussion

Following achievement of the low-prevalence goals for *S. mansoni*, halting MDA will very likely lead to the resurgence of infection unless prevalence is very low [12]. Therefore, some level of treatment is required to push prevalence back below these levels and maintain the goals. Our analyses suggest that the probability of maintaining the goals depends on the epidemiological setting (baseline prevalence [transmission potential] and the age intensity profile of infection), the goal, and the maintenance strategy adopted.

The choice of whether to maintain the goals after they are achieved or move towards the next target depends on the goal in question and the transmission setting. In moderate-transmission settings, particularly where there is a low adult burden of infection, switching to a maintenance strategy after achieving the morbidity control goal is likely to eventually lead to the achievement of the EPHP goal. If, however, the premaintenance strategy was continued for a few more years, the EPHP goal would be reached earlier, while simultaneously maintaining the morbidity control goal. Furthermore, regardless of the burden of infection in adults, in high-transmission settings with baseline SAC above *C_l_* (76% and 67% in SCHISTOX and ICL, respectively), there is a greater likelihood of maintaining the EPHP goal than the morbidity control goal. This is because, in these settings, the lower the prevalence when yearly MDA is halted, the lower the chances of resurgence if control efforts are reduced after reaching the morbidity goal. In such settings, it would be more feasible to move towards achieving and maintaining the EPHP goal. Generally, after the EPHP goal is reached, the likelihood of maintaining it depends on the transmission setting and strategy adopted, with more efforts required to keep the heavy-intensity prevalence in SAC below the threshold in high-transmission settings.

**Figure 3.**
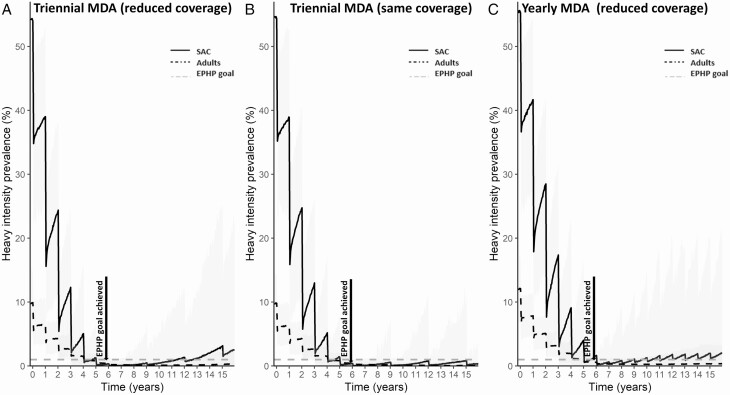
Prevalence of heavy-intensity infections in SAC and adults for a high-transmission setting (77% baseline SAC prevalence) with a low adult burden of infection. Plots are shown for achieving and maintaining the EPHP goal. EPHP is achieved after 7 years of annual treatment of 90% of SAC and 45% of adults and the maintenance strategies—(*A*) triennial MDA (reduced coverage strategy [50% SAC coverage]), (*B*) triennial MDA (same coverage required to reach the goal [90% of SAC and 45% of adults]), and (*C*) yearly MDA (reduced coverage strategy [50% SAC coverage])—are employed. Results shown are generated using SCHISTOX. Abbreviations: EPHP, elimination as a public health problem; MDA, mass drug administration; SAC, school-aged children.

Although our results suggest that less frequent or lower coverage MDAs (compared with what is required to reach the goals) could lead to a high likelihood of maintaining the goals, implementing these would require some thought and context-specific adaptation. The choice of what maintenance strategy to adopt depends on the goal to be maintained and the transmission setting. In moderate-transmission settings, regardless of the burden of infection in adults, both goals can be maintained employing any of the 3 strategies modeled here. Conversely, in high-transmission settings, the strategies that employ a reduced coverage (compared with that required to reach the goals) may not be sufficient to maintain the goals even if yearly MDA is continued after the goals are achieved. For example, regardless of the burden of infection in adults, in high-transmission settings above the cutoff baseline SAC prevalence, the yearly MDA strategy is able to maintain the goals only when a coverage close to that which was used to reach the goals is adopted (Figure 2). In these settings, after the goals are achieved, adopting a triennial MDA strategy and returning to administer mass treatment at the same coverage used to achieve the goal is more effective than continuing MDA with 50% SAC coverage. We must point out that, with the triennial MDA strategy, in the years when there is no MDA, the heavy-intensity SAC prevalence may go above the threshold (Figure 3). For these programs, the availability of PZQ in health facilities may enable infected individuals to have access to treatment and thus reduce the risk of resurgence above the threshold. Regardless of the maintenance strategy employed, complementary interventions such as improving water and sanitation hygiene [22], the availability of a vaccine [15, 23], and the incorporation of snail control [24] would be beneficial in addition to PZQ-based MDA.

The length of the maintenance program employed in this study could be flexible depending on the availability of resources. For example, the Schistosomiasis Consortium for Operational Research and Evaluation (SCORE) sustaining control studies, were carried out for a period of 5 years [25], although in these studies, either goal had been achieved prior to the start of the study. The WHO-recommended treatment coverage is at least 75% of SAC for MDA programs and, while in some school-based programs coverage levels up to 90% of SAC have been recorded [26], true coverage of each age group might widely fluctuate in the real world due to a variety of reasons [27]. Consequently, for the reduced treatment coverage strategies investigated in this study, adopting coverage levels as low as 50% in an effective program may involve randomly not providing treatment to a proportion of the population, which may be unethical. However, for long-running programs like the ones investigated here, there may be program fatigue, which could lead to low treatment coverages.

It is important to note that our analysis has assumed that coverage at each round of treatment is at random and that there is no migration. However, if a proportion of the population persistently do not adhere/have access to treatment or there is immigration of infected individuals from neighboring communities where infection may persist, they may serve as a reservoir of infection, thereby reducing the effectiveness of treatment programs. Additionally, the current WHO definitions of the morbidity control and EPHP goals based on heavy-intensity SAC prevalence may be inappropriate for determining success related to changes in infection as prevalence and morbidity may still be high even after the goals are achieved [28]. Consequently, further work is needed to redefine targets that are evidence based. We also note that, in this study, we have focused our analysis on *S. mansoni* but future analyses will be extended to *S. haematobium*. We have used 2 independently developed, individual-based models of schistosomiasis transmission and both show similar results in terms of what is required to maintain schistosomiasis at low prevalence levels. Using the same assumptions as we have in this study, other stochastic models [29, 30] would probably suggest similar results.

### Conclusions

There is a risk of resurgence if MDA is stopped after the goals are achieved in high-transmission settings, and our analysis suggests that the higher the prevalence at the time of stopping, the higher the risk of recrudescence. Our analysis suggests that triennial treatment offers a potential maintenance strategy for EPHP in moderate-transmission settings but would require careful monitoring as faster than expected resurgence could lead to increased morbidity. As there are no clear guidelines for the next steps after the goals are achieved, we hope that this work will stimulate discussions on this topic.

## Supplementary Data

Supplementary materials are available at *Clinical Infectious Diseases* online. Consisting of data provided by the authors to benefit the reader, the posted materials are not copyedited and are the sole responsibility of the authors, so questions or comments should be addressed to the corresponding author.

ciab246_suppl_Supplementary-Material

## Notes

***Supplement sponsorship.*** This article appears as part of the supplement “Sustainable control of neglected tropical diseases – surveillance and diagnostics”, sponsored by the NTD Modelling Consortium.

***Disclaimer.*** The views, opinions, assumptions, or any other information set out in this article are solely those of the authors and should not be attributed to the funders or any other person connected with the funders.

***Financial support.*** This work was supported by the Bill and Melinda Gates Foundation (grant number OPP1184344) through the NTD Modelling Consortium.

***Potential conflicts of interest.*** R. M. A. reports holding shares in GlaxoSmithKline, outside the submitted work. All other authors report no potential conflicts. All authors have submitted the ICMJE Form for Disclosure of Potential Conflicts of Interest. Conflicts that the editors consider relevant to the content of the manuscript have been disclosed.

## References

[1] World Health Organization. Weekly epidemiological record. Geneva, Switzerland: World Health Organization, 2019.

[2] Verjee MA . Schistosomiasis: still a cause of significant morbidity and mortality. Res Rep Trop Med 2019; 10:153–63. 32099508 10.2147/RRTM.S204345 PMC6997417

[3] World Health Organization. Schistosomiasis: progress report 2001–2011, strategic plan 2012–2020. Geneva, Switzerland: World Health Organization, 2013.

[4] Katz N , Chaves A, Pellegrino J. A simple device for quantitative stool thick-smear technique in Schistosomiasis mansoni. Rev Inst Med Trop Sao Paulo 1972; 14:397–400. 4675644

[5] World Health Organization. Introduction to NTD roadmap 2030. Geneva, Switzerland: World Health Organization, 2020.

[6] Montresor A , Crompton DW, Gyorkos TW, Savioli L. Helminth control in school-age children: a guide for managers of control programmes. Geneva, Switzerland: World Health Organization, 2002.

[7] Ismail M . Preventive chemotherapy in human helminthiasis, coordinating use of anthelminthic drugs in control interventions: a manual for health professionals and programme managers. IJMR 2007; 126:235.

[8] World Health Organization. Schistosomiasis-progress report 2001–2011 and strategic plan 2012–2020. Geneva, Switzerland: World Health Organization, 2013. Google Scholar, 2020:1–80. Available at: https://apps.who.int/iris/handle/10665/78074. Accessed 27 May 2021.

[9] Rollinson D , Knopp S, Levitz S, et al. Time to set the agenda for schistosomiasis elimination. Acta Trop 2013; 128:423–40. 22580511 10.1016/j.actatropica.2012.04.013

[10] Deol AK , Fleming FM, Calvo-Urbano B, et al. Schistosomiasis—assessing progress toward the 2020 and 2025 global goals. N Engl J Med 2019; 381:2519–28. 31881138 10.1056/NEJMoa1812165 PMC6785807

[11] Tchuem Tchuenté LA , Rollinson D, Stothard JR, Molyneux D. Moving from control to elimination of schistosomiasis in sub-Saharan Africa: time to change and adapt strategies. Infect Dis Poverty 2017; 6:42. 28219412 10.1186/s40249-017-0256-8 PMC5319063

[12] NTD Modelling Consortium Schistosomiasis Group. Insights from quantitative and mathematical modelling on the proposed WHO 2030 goal for schistosomiasis. Gates Open Res 2019; 3:1517. 10.12688/gatesopenres.13052.1 PMC6820450 31701091

[13] Anderson RM , May RM. Helminth infections of humans: mathematical models, population dynamics, and control. Adv Parasitol 1985; 24:1–101. 3904343 10.1016/s0065-308x(08)60561-8

[14] Anderson RM , Turner HC, Farrell SH, Truscott JE. Studies of the transmission dynamics, mathematical model development and the control of schistosome parasites by mass drug administration in human communities. Adv Parasitol 2016; 94:199–246. 27756455 10.1016/bs.apar.2016.06.003

[15] Kura K , Truscott JE, Toor J, Anderson RM. Modelling the impact of a Schistosoma mansoni vaccine and mass drug administration to achieve morbidity control and transmission elimination. PLoS Negl Trop Dis 2019; 13:e0007349. 31166956 10.1371/journal.pntd.0007349 PMC6550388

[16] Graham M , Ayabina D, Lucas TC, et al. SCHISTOX: an individual based model for the epidemiology and control of schistosomiasis. Infect Dis Model 2021; 6:438–47. 33665519 10.1016/j.idm.2021.01.010 PMC7897994

[17] Turner HC , Truscott JE, Bettis AA, et al. Evaluating the variation in the projected benefit of community-wide mass treatment for schistosomiasis: implications for future economic evaluations. Parasit Vectors 2017; 10:213. 28454578 10.1186/s13071-017-2141-5 PMC5410074

[18] Toor J , Alsallaq R, Truscott JE, et al. Are we on our way to achieving the 2020 goals for schistosomiasis morbidity control using current World Health Organization guidelines? Clin Infect Dis 2018; 66:S245–52. 29860290 10.1093/cid/ciy001 PMC5982704

[19] Toor J , Rollinson D, Turner HC, et al. Achieving elimination as a public health problem for *Schistosoma mansoni* and *S. haematobium*: when is community-wide treatment required? J Infect Dis 2020; 221:525–30. 10.1093/infdis/jiz609 PMC7289541 31829414

[20] Dyson L , Stolk WA, Farrell SH, Hollingsworth TD. Measuring and modelling the effects of systematic non-adherence to mass drug administration. Epidemics 2017; 18:56–66. 28279457 10.1016/j.epidem.2017.02.002 PMC5340860

[21] Toor J , Turner HC, Truscott JE, et al. The design of schistosomiasis monitoring and evaluation programmes: the importance of collecting adult data to inform treatment strategies for Schistosoma mansoni. PLoS Negl Trop Dis 2018; 12:e0006717. 30296257 10.1371/journal.pntd.0006717 PMC6175503

[22] Campbell SJ , Savage GB, Gray DJ, et al. Water, sanitation, and hygiene (WASH): a critical component for sustainable soil-transmitted helminth and schistosomiasis control. PLoS Negl Trop Dis 2014; 8:e2651. 24722335 10.1371/journal.pntd.0002651 PMC3983087

[23] Kura K , Collyer BS, Toor J, et al. Policy implications of the potential use of a novel vaccine to prevent infection with Schistosoma mansoni with or without mass drug administration. Vaccine 2020; 38:4379–86. 32418795 10.1016/j.vaccine.2020.04.078 PMC7273196

[24] World Health Organization. Schistosomiasis elimination: refocusing on snail control to sustain progress Available at: https://www.who.int/news/item/25-03-2020-schistosomiasis-elimination-refocusing-on-snail-control-to-sustain-progress. Accessed 27 May 2021.

[25] Ezeamama AE , He CL, Shen Y, et al. Gaining and sustaining schistosomiasis control: study protocol and baseline data prior to different treatment strategies in five African countries. BMC Infect Dis 2016; 16:229. 27230666 10.1186/s12879-016-1575-2 PMC4880878

[26] Mduluza T , Jones C, Osakunor DNM, et al. Six rounds of annual praziquantel treatment during a national helminth control program significantly reduced schistosome infection and morbidity levels in a cohort of schoolchildren in Zimbabwe. PLoS Negl Trop Dis 2020; 14:e0008388. 32569278 10.1371/journal.pntd.0008388 PMC7332090

[27] Li EY , Gurarie D, Lo NC, Zhu X, King CH. Improving public health control of schistosomiasis with a modified WHO strategy: a model-based comparison study. Lancet Glob Health 2019; 7:e1414–22. 31537371 10.1016/S2214-109X(19)30346-8 PMC7024988

[28] Colley DG , Fleming FM, Matendechero SH, et al. Contributions of the Schistosomiasis Consortium for Operational Research and Evaluation (SCORE) to schistosomiasis control and elimination: key findings and messages for future goals, thresholds, and operational research. Am J Trop Med Hyg 2020; 103.1_Suppl (2020): 125–34. 32400345 10.4269/ajtmh.19-0787 PMC7351304

[29] Collyer BS , Turner HC, Hollingsworth TD, Keeling MJ. Vaccination or mass drug administration against schistosomiasis: a hypothetical cost-effectiveness modelling comparison. Parasites Vectors 2019; 12:1–14. 31647019 10.1186/s13071-019-3749-4 PMC6813092

[30] Lo NC , Bogoch II, Blackburn BG, et al. Comparison of community-wide, integrated mass drug administration strategies for schistosomiasis and soil-transmitted helminthiasis: a cost-effectiveness modelling study. Lancet Glob Health 2015; 3:e629–38. 26385302 10.1016/S2214-109X(15)00047-9

