# Supplementary material for: Maintaining Low Prevalence of Schistosoma mansoni: Modeling the Effect of Less Frequent Treatment
Source: Clin Infect Dis. 2021 Jun 14;72(Suppl 3):S140–5. doi: 10.1093/cid/ciab246 (PMC8201569; doi:10.1093/cid/ciab246)
Supplement: ciab246_suppl_Supplementary-Material [file ciab246_suppl_supplementary-material.docx]

Supplementary File

| Parameter | SCHISTOX | ICL | Reference |
| --- | --- | --- | --- |
| Fecundity $\left( \lambda\right)$ | 0.34 eggs/female/sample | 0.34 eggs/female/sample | [1-3] |
| Aggregation parameter$(\alpha, k)$ | 0.04-0.24 | 0.04-0.24 | [4, 5] |
| Density dependent fecundity | 0.0007/female worm | 0.0007/female worm | [5, 6] |
| Worm life span | 5.7 years | 5.7 years | [1, 5, 7] |
| Low adult burden setting:  Age specific contact rates for 0-5, 5-10, 10-16, 16+ years old | 0.01, 1.2, 1, 0.02 | 0.01, 1.2, 1, 0.02 | [8, 9] |
| High adult burden setting:  Age specific contact rates for  0-5, 5-12, 12-20, 20+ years old | 0.01, 0.61, 1, 0.12 | 0.01, 0.61, 1, 0.12 | [8, 9] |
| Drug efficacy | 86.3% | 86.3% | [10] |
| Contact rate $(\beta)$ | 0.03-0.18 | - | - |
| Reproduction number $(R_{0})$ | - |  | - |
| Population size | 500 | 500 | - |

**Table S1:** Parameter values for *S. mansoni*

**
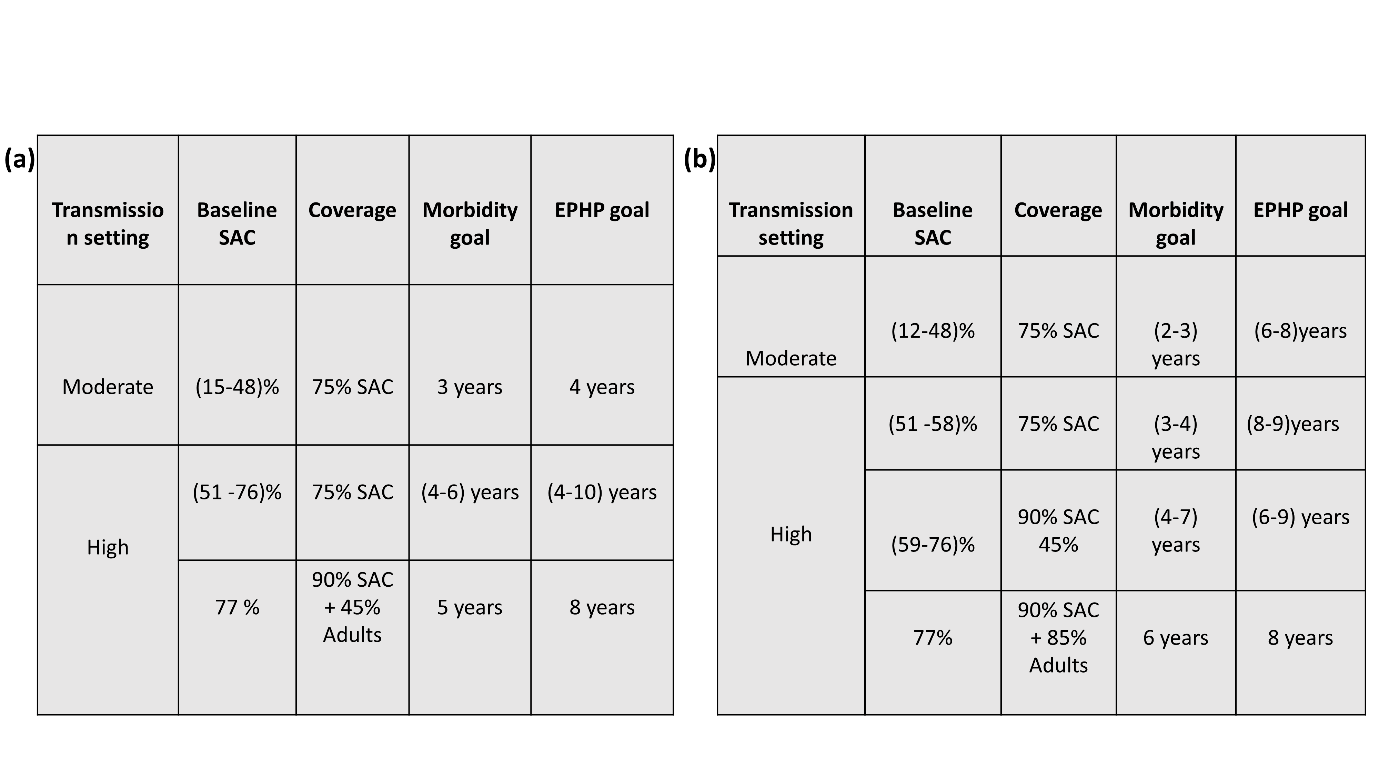
 Figure S1**: Number of years and coverage required to reach the morbidity control and EPHP goals for *Schistosoma mansoni* for (a) low and (b) high adult burden settings administering annual MDA. Results shown for SCHISTOX.


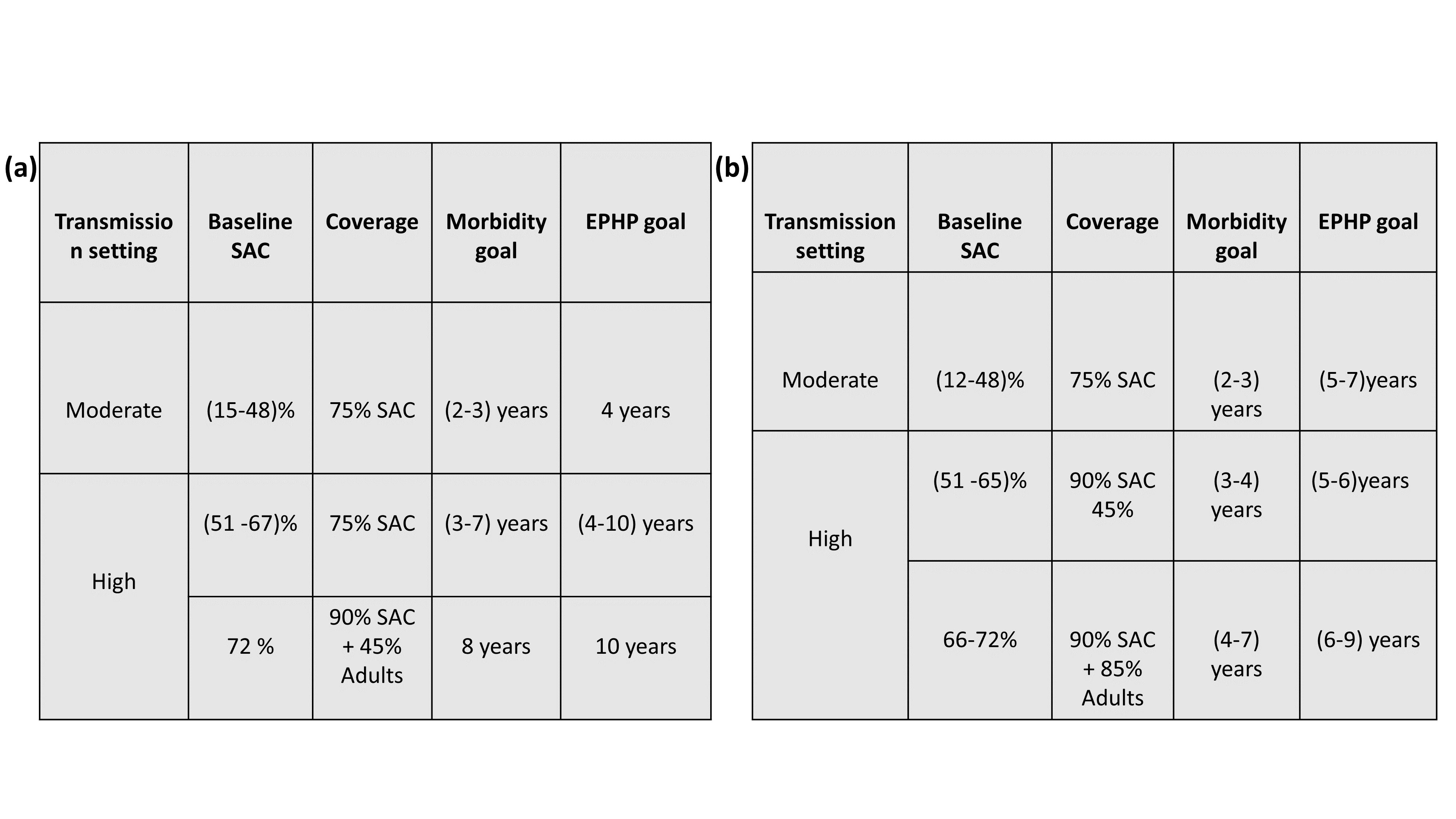
 **Figure S2**: Number of years and coverage required to reach the morbidity control and EPHP goals for *Schistosoma mansoni* for (a) low and (b) high adult burden settings administering annual MDA. Results shown for ICL.


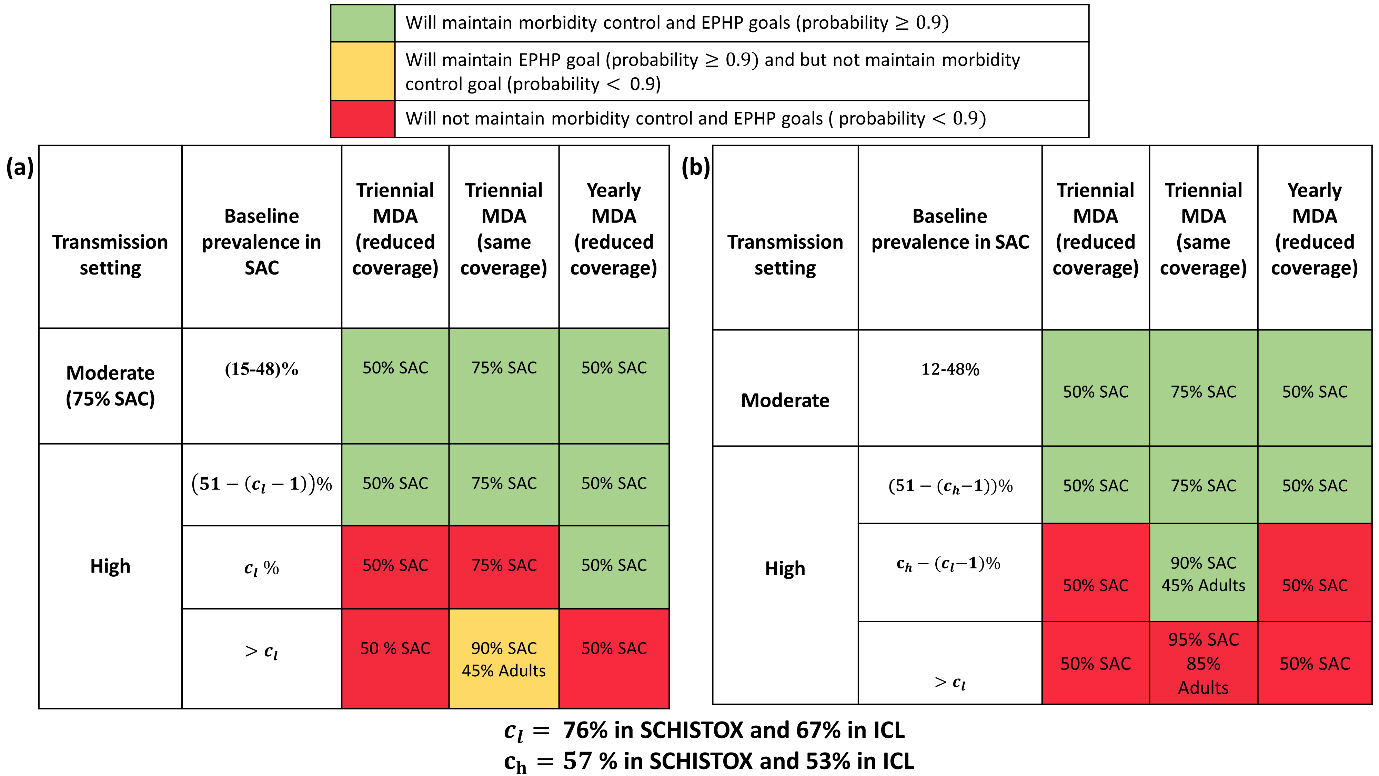


**Figure S3**:  Projected outcomes for *S. mansoni* employing different sustaining strategies for moderate to high transmission settings with (a) low and (b) high adult burden of infection with coverage levels used in each scenario. Results shown for both models.

**Table 2**: The Policy-Relevant Items for Reporting Models in Epidemiology of Neglected Tropical Diseases (PRIME-NTD) summary table [11].

| **Principle** | **What has been done to satisfy the principle?** | **Where in the manuscript is this described?** |
| --- | --- | --- |
| **Stakeholder engagement** | Reference to WHO guideline/roadmap | Introduction |
| **Complete model documentation** | Transmission models are briefly described in the manuscript and references to full description is provided. | Methods section |
| **Complete description of data used** | Parameters used are described in the manuscript | Table S1 |
| **Communicating uncertainty** | We have considered two age-intensity profiles for *S. mansoni* and different strategies to maintain the morbidity control and EPHP goals | Methods and Results sections |
| **Testable model outcomes** | The model outcomes can be tested | Discussion section |

**References**

1. Anderson RM, May RM. Population dynamics of human helminth infections: Control by chemotherapy. Nature **1982**; 297(5867): 557-63.

2. De Vlas S, Gryseels B, Van Oortmarssen G, Polderman A, Habbema J. A model for variations in single and repeated egg counts in Schistosoma mansoni infections. Parasitology **1992**; 104(3): 451-60.

3. De Vlas S, Nagelkerke N, Habbema J, Van Oortmarssen G. Review papers: statistical models for estimating prevalence and incidence of parasitic diseases. Statistical methods in medical research **1993**; 2(1): 3-21.

4. Truscott JE, Gurarie D, Alsallaq R, et al. A comparison of two mathematical models of the impact of mass drug administration on the transmission and control of schistosomiasis. Epidemics **2017**; 18: 29-37.

5. Anderson RM, Turner HC, Farrell SH, Truscott JE. Studies of the Transmission Dynamics, Mathematical Model Development and the Control of Schistosome Parasites by Mass Drug Administration in Human Communities. Advances in Parasitology **2016**; 94: 199-246.

6. Turner HC, Truscott JE, Bettis AA, et al. Evaluating the variation in the projected benefit of community-wide mass treatment for schistosomiasis: Implications for future economic evaluations. Parasites and Vectors **2017**; 10(1): 213-.

7. Fulford A, Butterworth A, Ouma J, Sturrock R. A statistical approach to schistosome population dynamics and estimation of the life-span of Schistosoma mansoni in man. Parasitology **1995**; 110(3): 307-16.

8. Turner HC, Truscott JE, Bettis AA, et al. Evaluating the variation in the projected benefit of community-wide mass treatment for schistosomiasis: Implications for future economic evaluations. Parasites & vectors **2017**; 10(1): 213.

9. Toor J, Turner HC, Truscott JE, et al. The design of schistosomiasis monitoring and evaluation programmes: The importance of collecting adult data to inform treatment strategies for Schistosoma mansoni. PLoS neglected tropical diseases **2018**; 12(10): e0006717.

10. Zwang J, Olliaro PL. Clinical efficacy and tolerability of praziquantel for intestinal and urinary schistosomiasis—a meta-analysis of comparative and non-comparative clinical trials. PLoS Negl Trop Dis **2014**; 8(11): e3286.

11. Behrend MR, Basáñez M-G, Hamley JI, et al. Modelling for policy: The five principles of the Neglected Tropical Diseases Modelling Consortium. PLoS neglected tropical diseases **2020**; 14(4): e0008033.
